# Clinical characteristics and risk factors of 47 cases with ruptured neuroblastoma in children

**DOI:** 10.1186/s12885-020-06720-9

**Published:** 2020-03-23

**Authors:** Hong Qin, Shen Yang, Siyu Cai, Qinghua Ren, Wei Han, Wei Yang, Haiyan Cheng, Xiaoli Ma, Huanmin Wang

**Affiliations:** 1 Department of Surgical Oncology, Beijing Children’s Hospital, Capital Medical University, National Center for Children’s Health, 56 Nanlishi Road, Beijing, 100045 China; 2 Center for Clinical Epidemiology & Evidence-Based Medicine, Beijing Children’s Hospital, Capital Medical University, National Center for Children’s Health, Beijing, 100045 China; 3 Hematology Oncology Center, Beijing Children’s Hospital, Capital Medical University, National Center for Children’s Health, 56 Nanlishi Road, Beijing, 100045 China

**Keywords:** Neuroblastoma, Tumor rupture, Clinical characteristics, Prognosis, Risk factors

## Abstract

**Background:**

Neuroblastoma (NB) tumor rupture is a rare oncology emergency with a poor prognosis. We aimed to evaluate patient clinical characteristics and risk factors for ruptured NB.

**Methods:**

A retrospective study of 47 patients with confirmed NB rupture between January 2009 and January 2019 at Beijing Children’s Hospital was conducted. To identify tumor rupture risk factors in high-risk NB patients, we included 93 consecutive non-ruptured high-risk NB patients from January 2017 to January 2019.

**Results:**

The median age at presentation was 29 months (adrenal and retroperitoneum origin) for 47 ruptured NB patients. Spontaneous tumor rupture occurred in 22 cases; 18 cases occurred during or after the first chemotherapy cycle, and 7 occurred after core needle biopsy. Five patients died of tumor rupture, and 17 patients’ parents refused further antitumor therapy. Among the 25 remaining patients, 6 survived without disease, 5 received ongoing treatment and achieved stable disease, and 14 died. According to multivariate logistic regression analysis, a maximum primary tumor diameter > 13.20 cm and *MYCN* gene amplification were independent risk factors for tumor rupture within high-risk NB.

**Conclusions:**

Tumor rupture is an uncommon, life-threatening event for NB patients; these patients are most likely to have poor outcomes due to tumor recurrence or rapid progression. Several treatment modalities, including symptomatic support therapy and chemotherapy, are important for saving lives and for developing NB risk-based treatment in the future.

## Background

Spontaneous tumor rupture in pediatric patients with neuroblastoma (NB) has been documented in previous case reports [1]. Tumor rupture is an uncommon, life-threatening presentation among NB patients, and several studies have reported that these patients have a poor prognosis [2]. Some patients are diagnosed with NB following spontaneous tumor rupture as the initial presentation. However, if the tumor is ruptured at initial presentation, accurate diagnosis may be difficult. In addition, some cases develop tumor rupture during or after chemotherapy and biopsy. Several treatment modalities have been described, including symptomatic supportive therapy, emergency or staged surgery, and chemotherapy. In this study, we retrospectively evaluated the clinical characteristics, treatment, and prognosis of ruptured NB cases. Moreover, to identify clinical risk factors for tumor rupture among NB patients, we compared the clinical characteristics between non-ruptured and ruptured NB. Thus, our goal was to contribute to the current knowledge of this rare disease and improve pre-existing treatment strategies.

## Methods

### Patient information

A total of 47 consecutive patients with ruptured NB who were diagnosed at Beijing Children’s Hospital (BCH) between January 2009 and January 2019 were included in this retrospective study. To compare the clinical characteristics between non-ruptured and ruptured high-risk NB, we included 93 consecutive patients with non-ruptured high-risk NB in the abdomen or pelvis from January 2017 to January 2019 in this retrospective study. Basic patient information was collected from the medical records. The initial diagnosis of NB was made according to International Neuroblastoma Staging System (INSS) criteria (unequivocal pathologic diagnosis was made from tumor tissue by light microscopy or bone marrow aspirate or trephine biopsy contained unequivocal tumor cells with increased urine/serum catecholamines/metabolites) [3]. However, no pathological review was performed in this study. In special cases of seriously ill patients without bone marrow metastasis, the initial clinical diagnosis was established by typical tumor localization with typical metastases (such as bone, liver, lymph node, and skin) detected by metaiodobenzylguanidine (MIBG) or fluorine-18-fluoro-2-deoxy-D-glucose positron emission tomography/computed tomography (^18^F-FDG PET/CT) combined with abnormal tumor marker levels. Patients were staged according to the International Neuroblastoma Risk Group Staging System (INRGSS) [4] and grouped by the INRG classification system [5]. The diagnostic criteria for tumor rupture were sudden abdominal pain, abdominal distension, anemia, and coagulation disorder that could not be explained by other reasons (bone marrow metastasis, bone marrow suppression after chemotherapy, and infection) and bloody ascites with or without finding an accurate location of tumor rupture by ultrasound and/or CT scan. Patients with ruptured NB were followed up to January 1, 2019. All methods were carried out in accordance with relevant guidelines and regulations, and the study was approved by the Medical Ethics Committee of Beijing Children’s Hospital (2017-k-89). A waiver of consent was awarded for the analyses conducted in this study.

### Laboratory analysis

Laboratory analysis was performed prior to treatment, and the interval between laboratory tests and biopsy was less than 15 days. Urinary vanillylmandelic acid (VMA) and homovanillic acid (HVA) were analyzed by gas chromatography-mass spectrometry (GC/MS), and their concentrations were expressed as a ratio to urinary creatinine concentration. Lactate dehydrogenase (LDH), neuron-specific enolase (NSE), and ferritin were measured in serum using routine clinical chemistry laboratory methods. Bone marrow metastatic disease was evaluated by bone marrow aspiration and biopsy. Tumors were classified in accordance with the International Neuroblastoma Pathology Classification System (INPC) [6]. In this study, *MYCN* gene copy numbers and segmental chromosome aberrations (1p and 11q) were analyzed using the fluorescence in situ hybridization (FISH) method.

### Treatment

Patients were treated with multimodal therapy based on the BCH-NB-2007 protocol for intermediate-risk NB combined with chemotherapy and surgery [7]. According to the biological features of the tumor, patients received 4 or 8 cycles of chemotherapy, which consisted of reduced doses of carboplatin and etoposide (CBVP) and cyclophosphamide, adriamycin and vincristine (CADO). In the BCH-NB-2007 protocol for high-risk NB (based on the Hong Kong N6 protocol), chemotherapy, surgery, and myeloablative therapy (carboplatin, etoposide and melphalan) were performed with autologous stem cell rescue, radiotherapy, and treatment of minimal residual disease with isotretinoin. Induction chemotherapy consisted of high-dose cyclophosphamide, adriamycin and vincristine (CAV) and high-dose cisplatinum and etoposide (CVP). Chemotherapy was performed every 21 days. Surgical resection of residual primary tumor or sites of regional dissemination (nodal disease) was performed after cycle 4, and peripheral blood stem cell harvesting was performed after cycle 5. Some patients underwent surgical resection of the primary tumor, and the extent of resection was defined as described in the report by Simon T et al. [8]. Gross total resection was defined as the removal of more than 90% of the tumor; macroscopically complete resection was defined as complete resection without macroscopic postoperative tumor residuals, which was confirmed by postoperative imaging studies.

### Statistical analysis

Statistical analysis was performed by SAS 9.4. Continuous variables were presented as the mean with standard deviation or median and interquartile range if the normality hypothesis test rejected the null hypothesis of normal distribution. Categorical variables were reported as counts and percentages. Two independent samples t-tests and *χ*^*2*^ tests were used to compare characteristics between the ruptured and non-ruptured groups. Receiver operating characteristic (ROC) curve analysis was performed to determine the most appropriate cut-off values. Univariate and multivariate logistic regression analyses were conducted to select potentially useful characteristics for predicting tumor rupture. Then, the area under the receiver operating characteristic (AUC-ROC) curves of the model were calculated. It should be noted that in this study, some tumor marker results were obtained after tumor rupture, as some patients were admitted to the hospital after spontaneous tumor rupture. Thus, the tumor marker results were not included in the analysis. *P* <  0.05 was considered statistically significant.

## Results

### Patient characteristics

During the period from January 2009 to January 2019, NB was diagnosed in approximately 1800 patients at our institute. A total of 47 ruptured NB patients (28 male and 19 female patients), with a median age at presentation of 29 months (range, 6 months to 8 years), were included in this study. Table 1 lists details regarding key patient characteristics. The median value of the maximum diameter of the primary tumor was 13.20 (10.99, 15.50) cm (range, 4.3 cm to 27.7 cm). Thirty-five patients (35/47, 74.47%) had INRG stage M disease, and metastatic sites included the bone marrow (22/35), bone (20/35), distant lymph nodes (19/35), liver (9/35), soft tissues (5/35), and brain (1/35).

**Table 1:** Clinical characteristics of 47 patients with neuroblastoma tumor rupture

Variables		Results ^1,2^
Gender	Female	19 (40.43)
	Male	28 (59.57)
Age (months)		29 (24, 46)
Primary site	Adrenal	29 (61.70)
	Retroperitoneum	18 (38.30)
NSE (ng/mL) ^3^	≤ 370	8 (17.02)
	> 370	39 (82.98)
Ferritin (ng/mL)		261.10 (161.70, 519.50)
LDH (U/L)		2978 (1772, 4120)
Urinary VMA (%)		17.04 (10.71, 72.06)
Urinary HVA (%)		17.70 (4.80, 32.45)
Maximum diameter of primary tumor (cm)		13.20 (10.99, 15.50)
INRG stage	L1	2 (4.26)
	L2	10 (21.28)
	M	35 (74.47)
	MS	0 (0.00)
INPC	Favorable	0 (0.00)
	Unfavorable	20 (100.00)
	Unknown	27
*MYCN* status	Not amplified	9 (31.03)
	Amplified	20 (68.97)
	Unknown	18
1p	Normal	8 (50.00)
	Aberration	8 (50.00)
	Unknown	31
11q	Normal	18 (85.71)
	Aberration	3 (14.29)
	Unknown	26
INRG risk	Very low	1 (2.38)
	Low	0 (0.00)
	Intermediate	1 (2.38)
	High	40 (95.24)
	Unknown	5

*NSE* neuron-specific enolase, *LDH* lactate dehydrogenase, *VMA* vanillylmandelic acid, *HVA* homovanillic acid, *INRG* International Neuroblastoma Risk Group, *INPC* International Neuroblastoma Pathology Classification

^1^Continuous variables are presented as median and interquartile range;

^2^Classification variables are presented as numbers (percent);

^3^Reference ranges of tumor markers: serum NSE ≤ 25 ng/mL; serum ferritin 6 ng/mL-159 ng/mL; serum LDH 110 U/L-295 U/L; urinary VMA 3.4–51.4%; urinary HVA 0.2–4.3%

### Tumor rupture

**Fig. 1:**
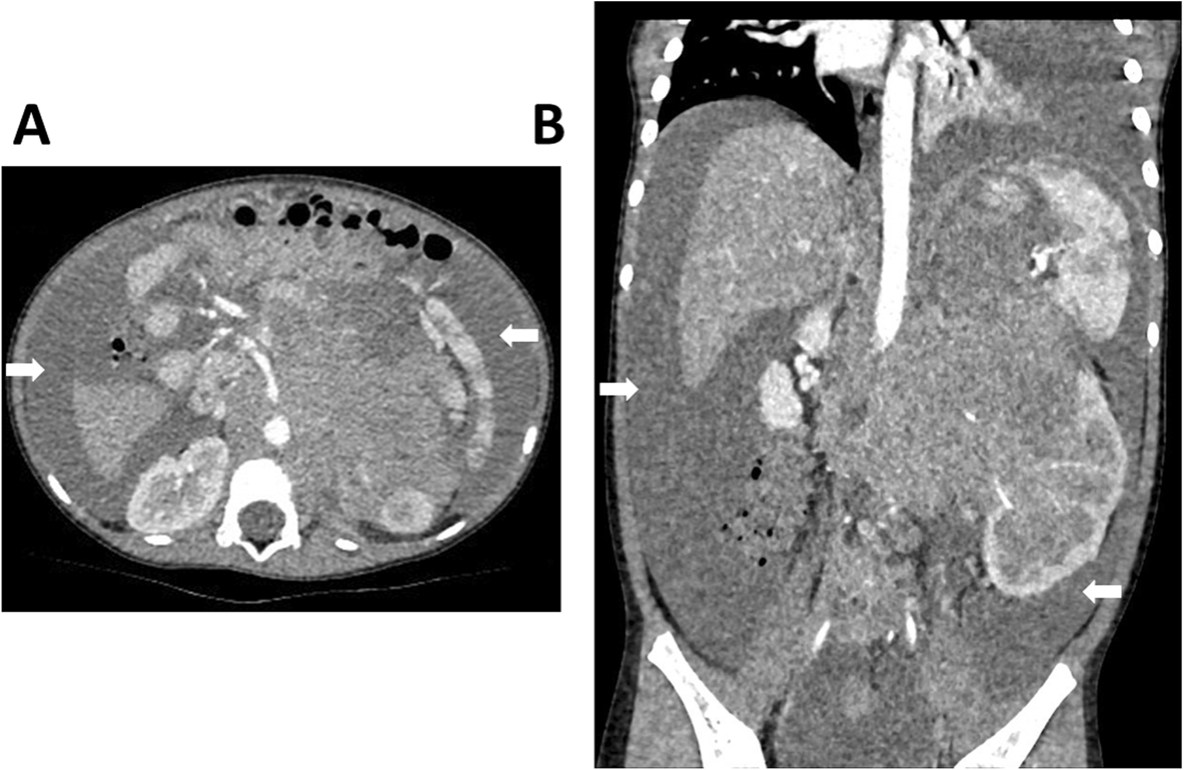
Abdominal enhanced computed tomography (CT) imaging findings of ruptured neuroblastoma. **a** Transverse section image. **b** Coronal reformatted image. The neuroblastoma in the left adrenal region is irregularly shaped, with no clear margins and hypodensity in the surrounding area, which is highly suggestive of tumor rupture (arrows denote the hypodense region)

**Fig. 2:**
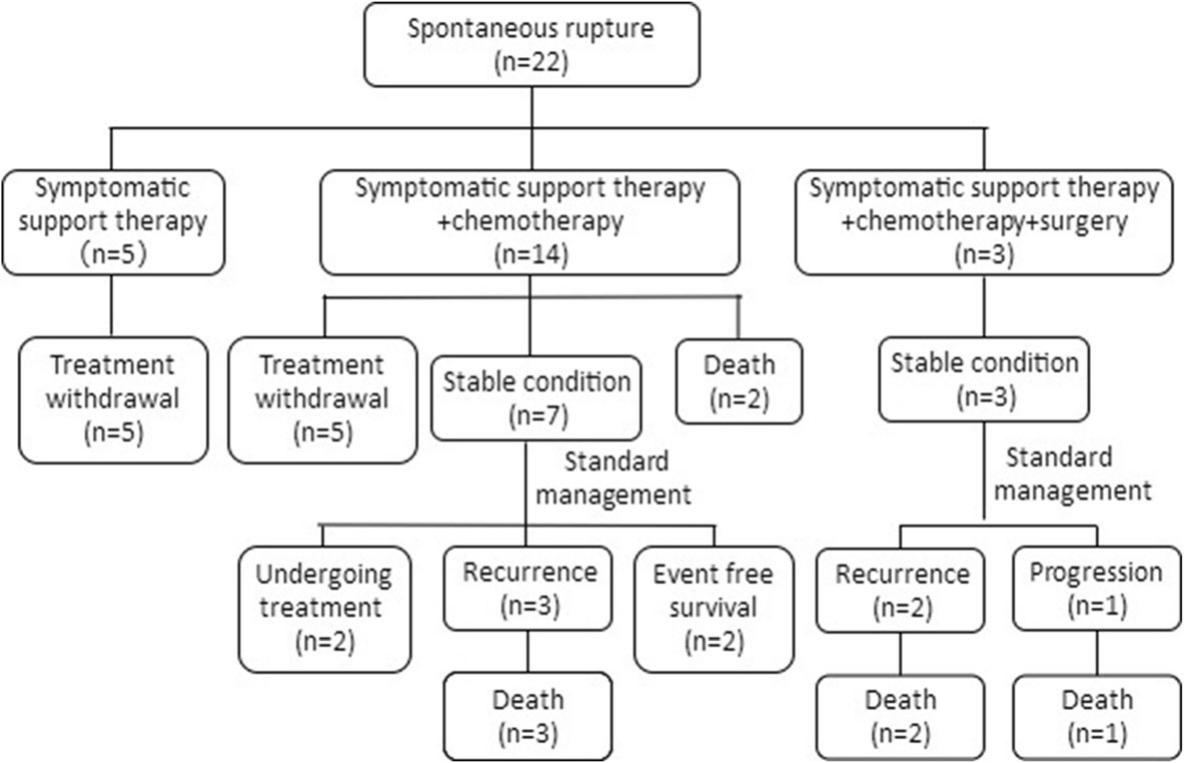
Treatment and prognosis of 22 patients with spontaneous NB rupture

**Fig. 3:**
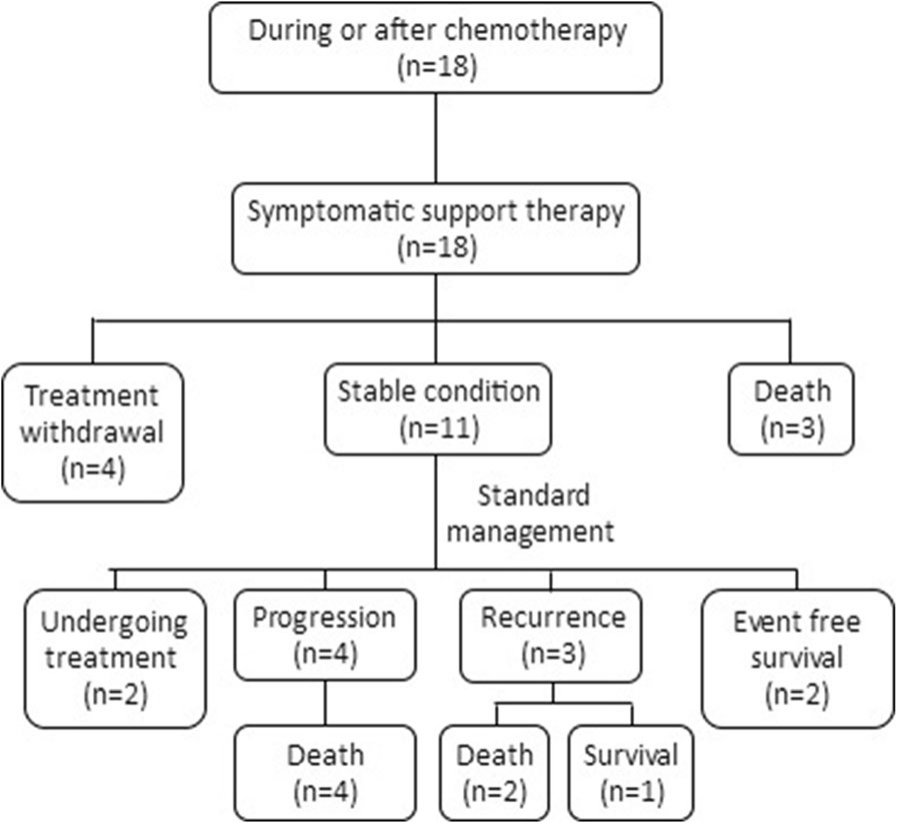
Treatment and prognosis of 18 patients with NB tumor rupture during or after chemotherapy

**Fig. 4:**
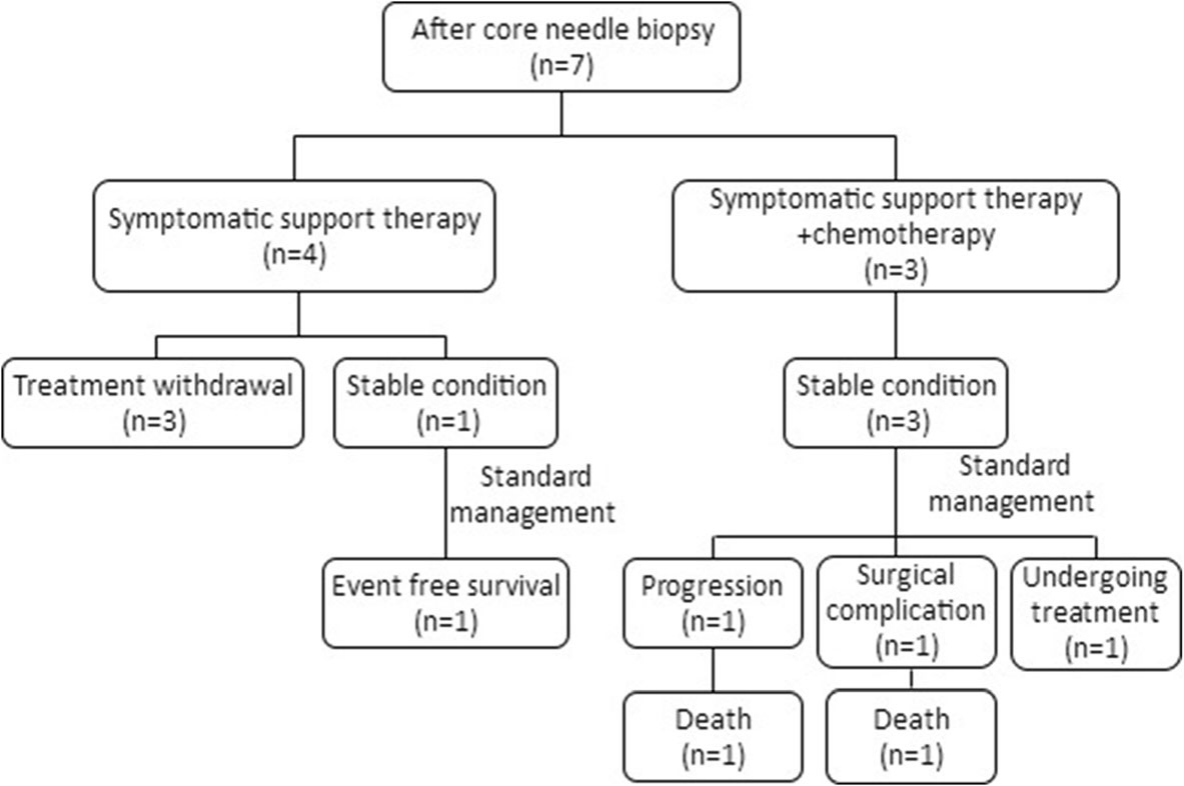
Treatment and prognosis of 7 patients with NB tumor rupture after core needle biopsy

Among the 47 ruptured NB patients, spontaneous tumor rupture occurred in 22 cases (46.81%); in 18 cases (38.30%), tumor rupture occurred during or after the first chemotherapy cycle (15 cases of CAV, 2 CBVP, and 1 CADO). From the first day of chemotherapy, the median time to rupture was 5 (2, 6) days. In another 7 cases (14.89%), tumor rupture occurred after core needle biopsy, with a median time to rupture of 6 (3, 7) days. Most patients experienced abdominal pain and abdominal distension and had a poor overall health status; all tumors were detected by ultrasound and/or CT scan (Fig. 1), and these patients were ultimately diagnosed with tumor rupture. The laboratory data revealed varying degrees of anemia in most patients, with a median hemoglobin level of 74 (58, 88) g/L (range, 36 g/L to 130 g/L). After receiving a diagnosis of tumor rupture, 5 patients (10.64%) received symptomatic supportive therapy with or without chemotherapy; all of these patients died of hemorrhagic shock, disseminated intravascular coagulation (DIC), and multiple organ dysfunction syndrome (MODS). Seventeen patients’ parents (36.17%) refused further therapy, and these patients were discharged in an unstable condition from the hospital against medical advice. The remaining 25 patients (53.19%) were discharged in a stable condition from the hospital after receiving symptomatic supportive therapy with or without chemotherapy and surgery. All 25 of these patients received further INRG risk-based therapy (Figs. 2, 3 and 4).

### Treatment

Of the 25 patients discharged in stable condition from the hospital, 23 (23/25, 92%) with high-risk NB received induction chemotherapy (CAV alternated with CVP), and 2 (2/25, 8%) with intermediate-risk NB received chemotherapy of CBVP alternated with CADO. Furthermore, 19 (19/25, 76%) underwent macroscopically complete resection of the primary tumor, 4 (4/25, 16%) underwent gross total resection (> 90%) of the primary tumor, and 2 (2/25, 8%) did not undergo resection surgery because of disease progression. Six patients received myeloablative therapy, autologous stem cell transplantation and further radiotherapy, while 9 received radiotherapy alone.

### Prognosis

In this study, 5 patients died of tumor rupture, and 17 patients’ parents refused any further antitumor therapy at our institute after the diagnosis of NB tumor rupture; these patients were lost to follow-up. Among the remaining 25 patients, 6 (6/25, 24%) survived until the end of follow-up (with survival times of 11 months, 17 months, 23 months, 32 months, 42 months, and 46 months), 5 (5/25, 20%) continued to receive treatment and achieved stable disease, and 14 (14/25, 56%) died (13 patients died of tumor recurrence or progression, and one died of renal failure after surgery), with a median survival time of 11 (7, 21) months (range, 2 months to 37 months).

In this study, 14 patients experienced tumor recurrence or progression, with a median time of 10 (6, 15) months (range, 2 months to 22 months) after diagnosis. Among these patients, 7 experienced tumor progression during therapy and ultimately died (4 cases of local progression and 3 cases of combined local and distant metastatic progression), while 7 experienced tumor recurrence (4 cases of local recurrence, one case of distant metastatic recurrence, and 2 cases of combined local and distant metastatic recurrence). Of these 7 patients, 6 died; only one patient survived, with a survival time of 46 months after chemotherapy and tumor resection.

### Tumor rupture risk factors

Since NB tumor rupture mainly occurs in children with high-risk NB (40/42, 95.24%), we further analyzed 93 cases of INRG high-risk NB patients with primary non-ruptured tumors in this study. By comparing the clinical characteristics between non-ruptured (*n* = 93) and ruptured (*n* = 40) high-risk NB (Table 2), we found significant differences in age, primary site, maximum diameter of the primary tumor, tumor marker levels, pathological characteristics, and the *MYCN* gene (*P* <  0.05).

**Table 2:** Comparison of clinical characteristics between ruptured and non-ruptured high-risk neuroblastoma groups

Variables		Non-ruptured neuroblastoma ^1,2^ (*n* = 93)	Ruptured neuroblastoma (*n* = 40)	Results ^3^	*P*
Gender	Female	39 (41.94)	16 (40.00)	0.043	0.8353
	Male	54 (58.06)	24 (60.00)		
Age (months)		43 (32, 59)	29 (24, 47)	−3.397	0.0007
Primary site	Adrenal	85 (91.40)	25 (62.50)	18.445	< 0.0001
	Retroperitoneum	7 (7.53)	15 (37.50)		
	Pelvic	1 (1.08)	0 (0.00)		
Maximum diameter of primary tumor (cm)		10.35 (7.30, 12.60)	13.60 (11.70, 16.00)	4.910	< 0.0001
Primary site of origin	Left	47 (50.54)	20 (50.00)	8.489	0.0143
	Right	42 (45.16)	12 (30.00)		
	Middle	4 (4.30)	8 (20.00)		
NSE (ng/mL) ^4^	≤ 370	44 (50.00)	4 (10.00)	18.773	< 0.0001
	> 370	44 (50.00)	36 (90.00)		
Ferritin (ng/mL)		232.40 (132.00, 481.15)	290.10 (207.30, 615.70)	1.448	0.1477
Urinary VMA (%)		191.99 (44.73, 537.60)	16.67 (10.56, 67.05)	−4.365	< 0.0001
Urinary HVA (%)		26.60 (12.74, 75.30)	17.70 (5.10, 32.13)	−2.135	0.0328
LDH (U/L)		723.00 (538.00, 1475.00)	3148.50 (2055.75, 4316.00)	6.912	< 0.0001
INPC categories	NB	58 (63.04)	25 (100.00)	19.823	< 0.0001
	GNBi	1 (1.09)	0 (0.00)		
	GNBn	33 (35.87)	0 (0.00)		
Grade of neuroblastic differentiation	Undifferentiated	1 (1.16)	1 (4.55)	4.603	0.1001
	Differentiating	24 (27.91)	2 (9.09)		
	Poorly differentiated	61 (70.93)	19 (86.36)		
MKI	< 2%	37 (49.33)	0 (0.00)	20.486	< 0.0001
	2–4%	28 (37.33)	11 (64.71)		
	> 4%	10 (13.33)	6 (35.29)		
INPC	Favorable	16 (19.75)	0 (0.00)	7.076	0.0078
	Unfavorable	65 (80.25)	18 (100.00)		
*MYCN* status	Not amplified	65 (73.03)	6 (23.08)	21.259	< 0.0001
	Amplified	24 (26.97)	20 (76.92)		
INRG stage	L1	1 (1.08)	1 (2.50)	6.090	0.1073
	L2	6 (6.45)	8 (20.00)		
	M	85 (91.40)	31 (77.50)		
	MS	1 (1.08)	0 (0.00)		

*NSE* neuron-specific enolase, *VMA* vanillylmandelic acid, *HVA* homovanillic acid, *LDH* lactate dehydrogenase, *INPC* International Neuroblastoma Pathology Classification, *NB* neuroblastoma, *GNBi* ganglioneuroblastoma, intermixed, *GNBn* ganglioneuroblastoma, nodular, *MKI* mitosis-karyorrhexis index, *INRG* International Neuroblastoma Risk Group

^1^Continuous variables are presented as the median and interquartile range;

^2^Classification variables are presented as numbers (percent);

^3^Results represent the z value of the Mann-Whitney test and the χ2 value of the chi-square test, respectively;

^4^Reference ranges of tumor markers: serum NSE ≤ 25 ng/mL; serum ferritin 6 ng/mL-159 ng/mL; urinary VMA 3.4–51.4%; urinary HVA 0.2–4.3%; serum LDH 110 U/L-295 U/L

In this study, some tumor marker results were obtained after tumor rupture, as some patients were admitted to the hospital after spontaneous tumor rupture. Thus, the tumor marker results were not included in the multivariate analysis. Ultimately, age, primary site, maximum diameter of the primary tumor, pathological characteristics (INPC categories, MKI, INPC), and *MYCN* gene were included in the multivariate logistic regression analysis. According to the maximum joint sensitivity and specificity values, the stratification value of age and maximum diameter of the primary tumor were calculated by ROC curve analyses. The cut-off values for the above characteristics were 29 months and 13.2 cm, respectively (Supplementary Figure 1).

In the multivariate logistic regression analysis, a maximum primary tumor diameter > 13.20 cm and *MYCN* gene amplification were two independent risk factors for high-risk NB tumor rupture, with adjusted odds ratios (ORs) of 6.401 (1.986, 20.626) and 7.874 (2.520, 24.603), respectively (Supplementary Table 1). The AUC-ROC of the model was 0.827, and the sensitivity and specificity were 96.2% (95% confidence interval: 78.4–99.8%) and 66.2% (95% confidence interval: 53.3–77.1%), respectively (Supplementary Figure 2).

As shown in Tables 1 and 2, *MYCN* amplification was detected in 69.0% (20/29) of ruptured NB patients and in 76.9% (20/26) of ruptured high-risk NB patients. A maximum primary tumor diameter > 13.20 cm was found in 48.9% (23/47) of ruptured NB patients and in 55.0% (22/40) of ruptured high-risk NB patients. Finally, the percentages of patients with *MYCN*-amplified tumors and tumors with diameters > 13.2 cm that had ruptured in the high-risk NB cohort (between January 2017 and January 2019) were 46.43% (13/28) and 34.38% (11/32), respectively.

## Discussion

Tumor rupture is an uncommon, life-threatening presentation among NB patients. Due to the rarity of NB tumor rupture, the previous literature mainly comprises case reports, while large-series case reports are lacking. To the best of our knowledge, the current case series of patients with NB tumor rupture is the largest reported series from a single institution to date. The results of this study have affirmed the following: 1) The main causes of NB tumor rupture include spontaneous rupture, tumor rupture during or after the first cycle of chemotherapy, and tumor rupture after core needle biopsy. 2) Tumor rupture occurs mostly in patients with high-risk NB. 3) After NB tumor rupture, symptomatic support treatment and chemotherapy are the main treatments, whereas surgery and interventional therapy are not usually the first choice. 4) NB tumor rupture is highly aggressive, disease progression or recurrence occurs early, and patients are susceptible to tumor recurrence with diffuse intraperitoneal lesions. 5) A maximum primary tumor diameter > 13.20 cm and *MYCN* gene amplification are independent risk factors for high-risk NB tumor rupture.

Spontaneous NB rupture is very rare in infants or children. This condition is more common in neonates, which can be explained by the trauma of delivery, especially when a congenital adrenal mass is crushed between the spine and liver [9–11]. Generally, the mechanism of spontaneous NB rupture is not fully understood. In terms of anatomic position, neonatal adrenal NB, which originates from the right side and is located between the spine and liver, is more prone to rupture [1, 12]. Regarding tumor size, a larger tumor is more likely to rupture. Previous reports have revealed that the risk of rupture is significantly increased when the maximum diameter of the tumor exceeds 10 cm [13, 14]. Regarding tumor components, tumors with solid components are less likely to rupture, while tumors with obvious cystic components and liquefaction necrosis are more likely to rupture. With regard to predisposing causes, some patients experience tumor rupture due to external forces such as trauma, delivery or tumor biopsy, while chemotherapy could induce tumor necrosis and might lead to altered blood flow to the capsule or surrounding tissue of the original tumor, resulting in coagulopathies that damage the tissue [12]. Since 2007, our institute has provided comprehensive treatment for NB, and during the study period (between January 2009 and January 2019), over 1000 children received chemotherapy, with only 18 patients experiencing tumor rupture (18/1000, 1.8%). Since 2013, our institute has carried out core needle biopsy for NB patients, and thus far, this procedure has been performed in more than 500 cases. In the present study, only 7 cases of tumor rupture were caused by core needle biopsy (7/500, 1.4%). However, except for age and INRG stage, no significant differences were found between the spontaneous and secondary (chemotherapy and core needle biopsy) NB rupture groups in terms of clinical characteristics or prognosis (Supplementary Table 2). Regarding the molecular biological characteristics of the tumor, 5 cases of spontaneously ruptured NB were reported in previous studies, and *MYCN* amplification was positive in 3 of 4 examined cases, suggesting that the aggressive behavior of *MYCN*-amplified NB predisposes the tumor to spontaneous rupture [1].

Previous studies have confirmed that *MYCN* gene amplification plays an important role in promoting angiogenesis and the proliferation, invasion, and metastasis of NB cells to inhibit cell differentiation and apoptosis [15, 16]. Targeting *MYCN* has significant potential for the treatment of highly vascularized NB. The blood vessel structure in malignant tumors is more fragile than that in normal tissues, which could cause infarction of the vessels and necrosis of the tumor capsule [16]. The above molecular biological basis is helpful in explaining the relationship between *MYCN* gene amplification and NB tumor rupture, but the specific mechanism requires further study.

The operative indications for spontaneous rupture of NB should be thoroughly considered. Evaluating imaging-defined risk factors (IDRFs) plays an important role in determining whether upfront surgery can be performed. For stable patients with resectable tumors (without IDRFs), complete resection is the best choice to ensure that the bleeding has stopped. In cases of unstable states or unresectable tumors (with IDRFs), interventional embolization or laparotomy for hemostasis as damage-control surgery might be considered. Interventional embolization is an effective treatment for tumors originating from organs such as the liver and kidney when spontaneous rupture occurs [17–20]. However, NB originates from the retroperitoneum and usually has no definitive blood supply. Thus, interventional embolization is typically ineffective. Considering the imaging characteristics of the patients in this study, most of the ruptured NB tumors were quite large. Additionally, the tumors were found to encase important intraperitoneal blood vessels and had already severely infiltrated adjacent organs or structures. Thus, IDRFs were present in most of the ruptured NBs, making upfront surgical resection extremely difficult. In this study, 3 patients with spontaneously ruptured NB underwent upfront surgery. During these operations, we found that these tumors were large, fragile and bled easily; they had also seriously invaded the adjacent organs and blood vessels. Therefore, appropriate surgical treatment must be determined according to the patient’s general condition in addition to the tumor features (such as INRG staging, origin, and local invasiveness). Exploration, hemostasis, and biopsy were the primary purposes if surgery was performed, and emergent tumor removal was unnecessary when hemostasis was achieved.

In terms of symptomatic support treatment, when tumor rupture occurred, vital signs were measured by monitoring ECG signals and recording urine volume, and supportive treatment was administered by providing oxygenation via inhalation and correcting shock via intravascular fluid therapy. Furthermore, blood samples were obtained for blood product preparation, and the patient was kept fully sedated and immobilized. Laboratory examinations and emergency imaging examinations should be performed immediately in such cases. According to the relevant tests and examinations, blood products such as erythrocytes, plasma, platelets and fibrinogen should be transfused to correct anemia, coagulation disorder and thrombocytopenia. Additionally, empirical anti-infective therapy, symptomatic myocardial protection, diuresis, correction of water and electrolyte disorders, and nutritional support therapy should be performed.

Imaging examinations, nuclear medical examinations, and laboratory examinations should be performed as soon as possible in order to initiate antitumor therapy. NB-related tumor markers, bone marrow aspiration and biopsy, MIBG or PET-CT, and cranial CT/MRI should also be performed to determine the tumor burden and stage. In addition, histopathological biopsy specimens of primary or metastatic lesions should be obtained as soon as the patient is stabilized. Molecular biology tests of the *MYCN* gene, 1p36, 11q23 and DNA ploidy should also be carried out. If a patient’s condition is too poor to receive general anesthesia and surgery, core needle aspiration biopsy under local anesthesia might be a suitable option to obtain tumor tissue with less stress on the patient. Through the above examinations, we determined the diagnosis of NB and performed INRG staging and risk stratification. Histological evidence could not be obtained for some patients who were highly clinically suspected of NB without bone marrow metastasis. In these extreme cases, the oncologists informed the patients’ parents that clinical diagnosis of NB and empirical chemotherapy were necessary life-saving procedures. However, once the patient is stable, pathological histology and molecular biology tests should be performed as soon as possible to correct NB staging and grouping as needed. To prevent tumor lysis syndrome, adequate hydration and alkalization should be ensured, as these aspects play important roles in controlling the tumor burden and improving the overall condition of the patient. For high-risk NB patients in very poor condition who cannot tolerate high-intensity chemotherapy, dose-induced chemotherapy could be performed during the first cycle of therapy, followed by standard protocols in the following cycles.

The results of this study showed that the prognosis of NB with tumor rupture was very poor. A few patients died directly due to MODS manifestations, such as hemorrhagic shock, heart failure, respiratory failure, and severe infection caused by tumor rupture. However, most patients were discharged in a stable condition after symptomatic support treatment and chemotherapy and received further stratified treatment according to their risk grouping. The conditions of most patients were stabilized by intensive preoperative induction chemotherapy; the levels of tumor markers decreased, tumors shrank, and metastatic disease was alleviated or disappeared. Although some patients have the opportunity for delayed surgery, most are susceptible to progression or early recurrence. The median time to progression or recurrence was 10 (6, 15) months in this study. Only one patient survived after tumor recurrence; all other patients died. Researchers analyzed the clinical and prognostic information of 2266 patients with NB recurrence or progression in the INRG database [21]. The median time to NB progression or recurrence was 13.2 months; the median time to recurrence was 11 months in 562 patients with *MYCN* amplification and 14.5 months in 1141 patients with no *MYCN* amplification, with a significant difference noted between the two groups (*P* <  0.05). The 5-year overall survival (OS) rate of 2266 patients with recurrence was only 20% ± 1%, and patients who relapsed between 6 and 18 months after diagnosis had the highest risk of death (the peak value was at approximately 12 months), which also supports the results of our study [21]. According to previous clinical studies, the most common recurrence sites in high-risk NB patients are bone and bone marrow, while the 5-year local recurrence rate of the primary site is only 11.9% ± 2.2% [22]. In this study, among 14 patients with disease progression or recurrence, 13 experienced intraperitoneal progression or recurrence; these patients often presented with diffuse intraperitoneal lesions, which strongly suggested that progression and recurrence were related to implant metastasis caused by tumor rupture.

## Conclusions

Tumor rupture is an uncommon, life-threatening presentation among NB patients, and patients with ruptured NB are most likely to have a poor outcome due to rapid progression or recurrence. Treatment modalities such as symptomatic support therapy and chemotherapy with/without emergency surgery are important for saving lives and for developing NB risk-based treatment strategies in the future. Additionally, a maximum primary tumor diameter > 13.20 cm and *MYCN* gene amplification are two independent risk factors for high-risk NB tumor rupture. Thus, we can predict tumor rupture early among NB patients and then plan to intervene as soon as possible, ultimately improving the prognosis of these patients.

## Supplementary information

**Additional file 1:Supplementary Figure 1.** ROC curve analyses. Stratification values for (A) age and (B) the maximum diameter of the primary tumor, which were calculated by ROC curve analyses.

**Additional file 2: Supplementary Figure 2.** ROC curve for the prediction of high-risk NB tumor rupture. A maximum primary tumor diameter > 13.20 cm and *MYCN* gene amplification were used to predict high-risk NB tumor rupture.

**Additional file 3: Supplementary Table 1.** Multivariate logistic regression analysis.

**Additional file 4: Supplementary Table 2.** Comparison of clinical characteristics and prognosis between different causes of ruptured neuroblastoma groups.

## Data Availability

All data generated or analyzed during this study are included in this published article and its supplementary information files.

## Abbreviations

AUC-ROC: Area under the receiver operating characteristic
BCH: Beijing Children’s Hospital
CADO: Cyclophosphamide, adriamycin and vincristine
CAV: Cyclophosphamide, adriamycin and vincristine
CBVP: Carboplatin and etoposide
CVP: Cisplatinum and etoposide
DIC: Disseminated intravascular coagulation
^18^F-FDG PET/CT: Fluorine-18-fluoro-2-deoxy-D-glucose positron emission tomography/computed tomography
FISH: Fluorescence in situ hybridization
GC/MS: Gas chromatography-mass spectrometry
HVA: Homovanillic acid
IDRFs: Imaging-defined risk factors
INPC: International Neuroblastoma Pathology Classification System
INRGSS: International Neuroblastoma Risk Group Staging System
INSS: International Neuroblastoma Staging System
LDH: Lactate dehydrogenase
MIBG: Metaiodobenzylguanidine
MODS: Multiple organ dysfunction syndrome
NB: Neuroblastoma
NSE: Neuron-specific enolase
ORs: Odds ratios
OS: Overall survival
ROC: Receiver operating characteristic
VMA: Vanillylmandelic acid
