# Supplementary figures and images for: Clinical characteristics and risk factors of 47 cases with ruptured neuroblastoma in children
Source: BMC Cancer. 2020 Mar 23;20:243. doi: 10.1186/s12885-020-06720-9 (PMC7092550; doi:10.1186/s12885-020-06720-9)

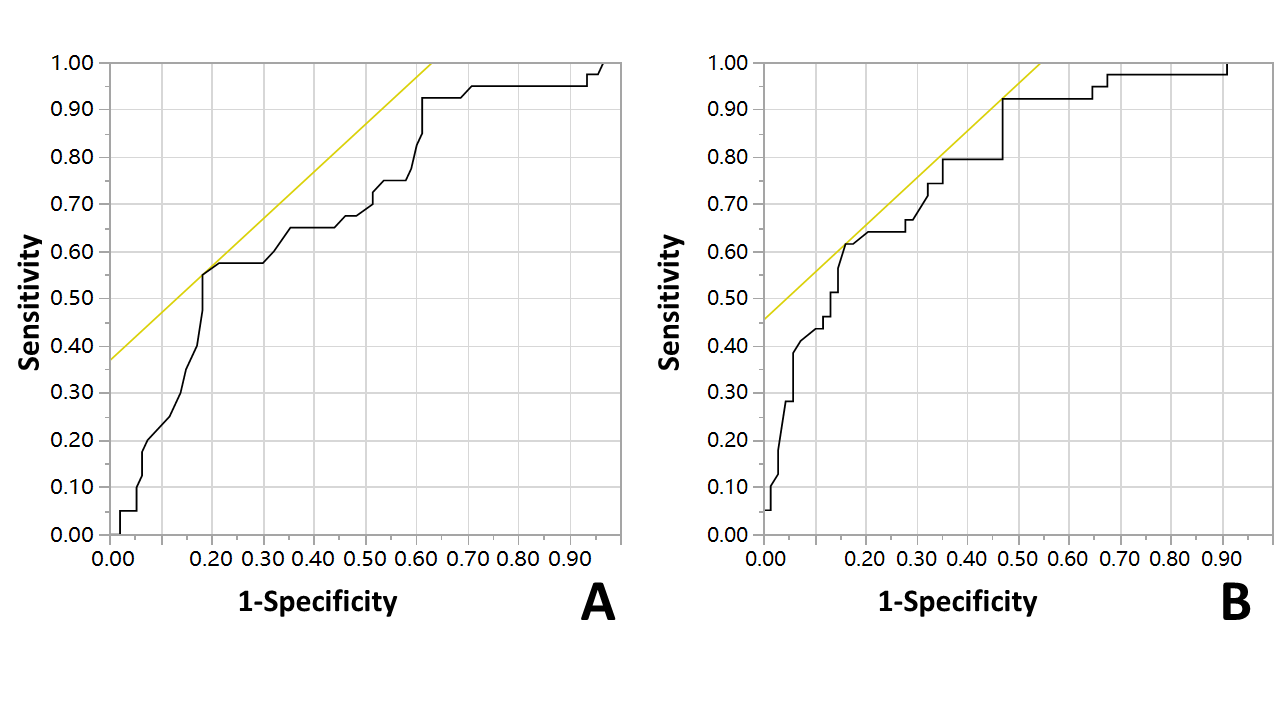

Supplement: Supplementary file 1 — Additional file 1:Supplementary Figure 1. ROC curve analyses. Stratification values for (A) age and (B) the maximum diameter of the primary tumor, which were calculated by ROC curve analyses. [file 12885_2020_6720_MOESM1_ESM.tif]

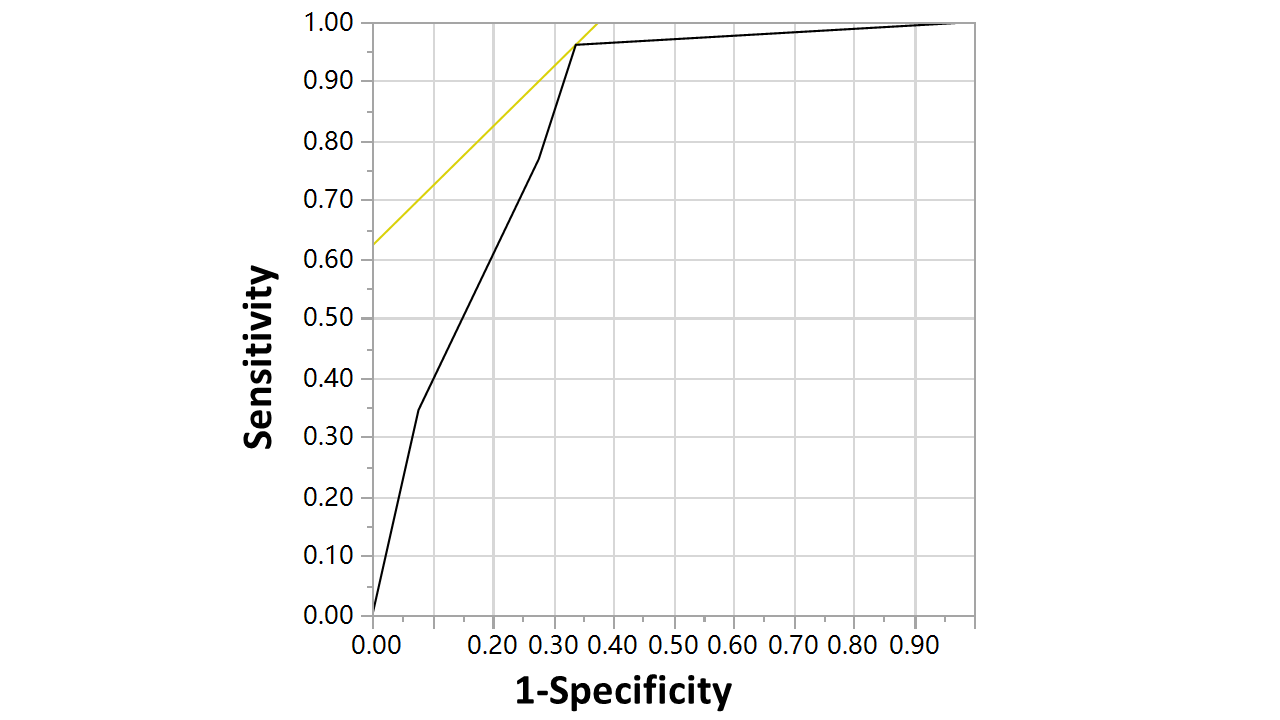

Supplement: Supplementary file 2 — Additional file 2: Supplementary Figure 2. ROC curve for the prediction of high-risk NB tumor rupture. A maximum primary tumor diameter > 13.20 cm and MYCN gene amplification were used to predict high-risk NB tumor rupture. [file 12885_2020_6720_MOESM2_ESM.tif]
