# Supplementary material for: Clinical characteristics and risk factors of 47 cases with ruptured neuroblastoma in children
Source: BMC Cancer. 2020 Mar 23;20:243. doi: 10.1186/s12885-020-06720-9 (PMC7092550; doi:10.1186/s12885-020-06720-9)
Supplement: Supplementary file 3 — Additional file 3: Supplementary Table 1. Multivariate logistic regression analysis. [file 12885_2020_6720_MOESM3_ESM.docx]

Supplementary Table 1. Multivariate logistic regression analysis

| Characteristics | Estimate | Standard error | Wald | *P* | OR |
| --- | --- | --- | --- | --- | --- |
| *MYCN* amplification | 2.064 | 0.581 | 12.602 | 0.0004 | 7.874 (2.520, 24.603) |
| maximum diameter of primary tumor > 13.20 cm | 1.856 | 0.597 | 9.668 | 0.0019 | 6.401 (1.986, 20.626) |

OR: odds ratio.
