# Supplementary material for: Clinical characteristics and risk factors of 47 cases with ruptured neuroblastoma in children
Source: BMC Cancer. 2020 Mar 23;20:243. doi: 10.1186/s12885-020-06720-9 (PMC7092550; doi:10.1186/s12885-020-06720-9)
Supplement: Supplementary file 4 — Additional file 4: Supplementary Table 2. Comparison of clinical characteristics and prognosis between different causes of ruptured neuroblastoma groups. [file 12885_2020_6720_MOESM4_ESM.docx]

Supplementary Table 2. Comparison of clinical characteristics and prognosis between different causes of ruptured neuroblastoma groups

| Variables | | Spontaneous (n = 22) ^1,2^ | Chemotherapy (n = 18) | Biopsy  (n = 7) | Results ^3^ | *P* |
| --- | --- | --- | --- | --- | --- | --- |
| Gender | Female | 8 | 9 | 2 | 1.251 | 0.5349 |
|  | Male | 14 | 9 | 5 |  |  |
| Age (months) |  | 39 (28, 48) | 27 (18, 30) | 25 (14, 44) | 7.795 | 0.0203 |
| Primary site | Adrenal | 11 | 14 | 4 | 3.429 | 0.1801 |
|  | Retroperitoneum | 11 | 4 | 3 |  |  |
| Maximum diameter of primary tumor (cm) |  | 13.70 (11.62, 15.80) | 12.85 (10.78, 16.10) | 12.20 (10.80, 13.30) | 0.991 | 0.6091 |
| NSE (ng/mL) ^4^ | ≤ 370 | 4 | 3 | 1 | 0.061 | 0.9699 |
|  | > 370 | 18 | 15 | 6 |  |  |
| Ferritin (ng/mL) |  | 356.90 (112.80, 702.60) | 245.30 (206.60, 412.13) | 330.75 (110.20, 751.30) | 0.283 | 0.8681 |
| Urinary VMA (%) |  | 14.37 (9.17, 234.12) | 39.68 (13.81, 81.17) | 14.18 (8.45, 72.06) | 1.034 | 0.5962 |
| Urinary HVA (%) |  | 14.65 (4.24, 45.86) | 24.70 (8.74, 45.27) | 11.39 (3.03, 19.55) | 3.165 | 0.2055 |
| LDH (U/L) |  | 2676 (1143, 4271) | 2685 (1658, 3902) | 3329 (2215, 5151) | 0.323 | 0.8509 |
| INPC categories | NB | 11 | 11 | 7 | - | - |
|  | GNBi | 0 | 0 | 0 |  |  |
|  | GNBn | 0 | 0 | 0 |  |  |
| Grade of neuroblastic differentiation | Undifferentiated | 0 | 0 | 1 | 6.885 | 0.1419 |
|  | Differentiating | 0 | 2 | 0 |  |  |
|  | Poorly differentiated | 9 | 7 | 6 |  |  |
| MKI | < 2% | 0 | 0 | 0 | 0.297 | 0.8614 |
|  | 2% - 4% | 5 | 6 | 2 |  |  |
|  | > 4% | 3 | 2 | 1 |  |  |
| INPC | Favorable | 0 | 0 | 0 | - | - |
|  | Unfavorable | 8 | 8 | 4 |  |  |
| *MYCN* status | Not amplified | 4 | 4 | 1 | 1.009 | 0.6039 |
|  | Amplified | 6 | 9 | 5 |  |  |
| 11q | Normal | 3 | 12 | 3 | 3.444 | 0.1787 |
|  | Aberration | 2 | 1 | 0 |  |  |
| 1p | Normal | 3 | 4 | 1 | 0.541 | 0.7629 |
|  | Aberration | 2 | 4 | 2 |  |  |
| INRG stage | L1 | 1 | 0 | 1 | 9.870 | 0.0427 |
|  | L2 | 8 | 2 | 0 |  |  |
|  | M | 13 | 16 | 6 |  |  |
|  | MS | 0 | 0 | 0 |  |  |
| INRG risk | Very low | 1 | 0 | 0 | 3.584 | 0.7328 |
|  | Low | 0 | 0 | 0 |  |  |
|  | Intermediate | 0 | 1 | 0 |  |  |
|  | High | 19 | 15 | 6 |  |  |
| Hemoglobin (g/L) |  | 78 (53, 96) | 72 (59, 87) | 74 (58, 80) | 0.179 | 0.9142 |
| Prognosis | Died of tumor rupture | 2 | 3 | 0 | 4.371 | 0.6266 |
|  | Treatment withdraw after tumor rupture | 10 | 4 | 3 |  |  |
|  | Survived after risk-based therapy | 4 | 5 | 2 |  |  |
|  | Died after risk-based therapy | 6 | 6 | 2 |  |  |

^1^ Continuous variables are presented as the median and interquartile range;

^2^ Classification variables are presented as numbers;

^3^ Results represent the z value of the Mann-Whitney test and the χ2 value of the chi-square test, respectively;

^4^ Reference ranges of tumor markers: serum NSE ≤ 25 ng/mL; serum ferritin 6 ng/mL-159 ng/mL; urinary VMA 3.4%-51.4%; urinary HVA 0.2%-4.3%; serum LDH 110 U/L-295 U/L.

NSE, neuron-specific enolase; VMA, vanillylmandelic acid; HVA, homovanillic acid; LDH, lactate dehydrogenase; INPC, International Neuroblastoma Pathology Classification; NB, neuroblastoma; GNBi, ganglioneuroblastoma, intermixed; GNBn, ganglioneuroblastoma, nodular; MKI, mitosis-karyorrhexis index; INRG, International Neuroblastoma Risk Group.
